# Breathlessness without borders: a call to action for global breathlessness research

**DOI:** 10.1038/s41533-024-00384-9

**Published:** 2024-09-30

**Authors:** Joseph David Clark, Kate Binnie, Maddie Bond, Michael Crooks, David C. Currow, Jordan Curry, Helen Elsey, Monsur Habib, Ann Hutchinson, Ireneous Soyiri, Miriam J. Johnson, Shreya Nair, Seema Rao, Noemia Siqueira-Filha, Anna Spathis, Siân Williams

**Affiliations:** 1 Wolfson Palliative Care Research Centre, University of Hull, Hull, UK; 2 Respiratory Research Group, Hull York Medical School, University of Hull and Hull University Teaching Hospitals NHS Trust, Hull, UK; 3 Graduate School of Medicine, Faculty of Science, Medicine and Health, University of Wollongong, Wollongong, NSW Australia; 4 Department of Health Sciences, University of York, York, UK; 5 Bangladesh Primary Care Respiratory Group, Khulna, Bangladesh; 6 Institute of Clinical and Applied Health Research, University of Hull, Hull, UK; 7 Department of Palliative Medicine and Supportive Care, Kasturba Medical College, Manipal Academy of Higher Education, Manipal, India; 8 Bangalore Hospice Trust, Bengaluru, India; 9 University of Cambridge, Cambridge, UK; 10 International Primary Care Respiratory Group, Edinburgh, UK

**Keywords:** Public health, Respiratory signs and symptoms, Palliative care

## Abstract

It is likely that the burden of breathlessness in low and middle-income countries (LMICs) is much higher than has been estimated using calculations of disease burden and expected prevalence of the symptom. However, most breathlessness research has been conducted in high-income countries and may not be relevant to LMICs. To address this issue, we convened an international breathlessness and global health workshop. Our multidisciplinary team of experts (global palliative care, respiratory medicine, epidemiology, palliative medicine, psychiatry, sport science, global public health and health economics) met at the University of Hull for a two-day workshop in May 2024. We had 8 presentations on key issues relevant to global breathlessness research. Our discussions focussed on unexplored questions and links between breathlessness and other health and social issues, in order to develop an agenda for global breathlessness research. Our discussions highlighted (1) the global burden of breathlessness generated by a range of lifestyle, environmental, disease and poverty-related factors, (2) the need for a global healthcare workforce that can address modifiable causes and the symptoms of breathlessness together using an integrated approach, (3) the value of information over clinical effectiveness when considering implementation of breathlessness self-management interventions, (4) Addressing non-clinical outcomes which are meaningful to individuals and families and (5) Developing a language for global breathlessness research which does not assume that the cause of breathlessness is diagnosed or treated. We present our discussions and recommendations for new approaches and paradigms for global breathlessness research to generate discussion—not to provide empirical evidence.

## Introduction

As life expectancies increase, more people live long enough to develop multiple long-term conditions. Healthcare systems worldwide are re-organising, to achieve universal health coverage with integrated person-centred care essential for meeting the needs of people with multimorbidity. Person-centred care requires equitable approaches between disease modification and symptom management, addressing the unique needs of the person and their family. However, although not mutually exclusive, symptom management is commonly assumed to be primarily addressed through disease modification rather than seen as a therapeutic target in its own right^1^, despite the serious negative impact of symptoms on individuals, families and health workers.

Daily breathlessness is breathlessness which occurs every day, causing distress and disability. Daily breathlessness is a prevalent symptom and chronic health problem that generates a high burden of suffering but often goes unaddressed. Daily breathlessness affects nearly all (>80%) of those with chronic obstructive pulmonary disease (COPD)^2^, advanced lung cancer^3^, and advanced heart failure^4^. It is also common in people with anxiety and obesity. Crucially, daily breathlessness may have multiple causes, which may or may not, be optimally managed and incorporate all sub-sets of long-term breathless populations (e.g. refractory breathlessness, chronic breathlessness, etc). For people living with multiple long-term conditions, breathlessness is almost certain, driving self-limitation of activity, reduction in fitness and social and human capital. Treating the symptom of breathlessness alongside disease modification is consistent with patient and family preferences. Daily breathlessness results in poorer physical and mental health, ability to work, and increased care needs and health expenditure. Despite these broad consequences of breathlessness, most breathlessness research has evaluated changes in quality-of-life outcomes in the context of advanced illness only. Little research has explored breathlessness as a societal problem, or how addressing breathlessness may be able to improve broader health and socioeconomic outcomes, like regular exercise or going to work.

Clinical practice frameworks which promote self-efficacy—the active involvement of the patient in their own care—are increasingly recognised in high-income countries (HICs) and increase patient control over their own behaviours to improve their healthcare outcomes^5^. However, although most people with daily breathlessness live in low and middle-income countries (LMICs)^6^, where self-efficacy approaches are not in routine use, most breathlessness research takes place in HICs. The need to understand context is fundamental for the design and delivery of healthcare which addresses local needs^7^. Yet, breathlessness research has not yet explored how social norms, environmental factors (e.g. heat, humidity, air pollution) and other contextual factors may influence how the problem of breathlessness is understood and how it may be helped. Furthermore, most breathlessness research in HICs is predicated on optimal management of the underlying cause (e.g. chronic breathlessness syndrome research), whereas daily breathlessness may have multiple causes which may never be diagnosed or treated. In relation to these challenges, we convened a two-day Breathlessness and Global Health Research workshop to discuss breathlessness in the global context and to establish an agenda for global breathlessness research.

## Breathlessness and global health research workshop

We held a hybrid workshop at the University of Hull, United Kingdom, on 21st and 22nd May 2024. Sixteen people, from five countries (United Kingdom, India, Bangladesh, Brazil, Ghana) attended contributing a range of academic and clinical experiences of breathlessness (global palliative care, respiratory medicine, epidemiology, palliative medicine, psychiatry, exercise science, global public health, and health economics). Attendees were invited from within our networks, because of their areas of expertise, ongoing collaborations with the organisers and interest in breathlessness as a global health inequality. Across two days, eight presentations addressed topics relevant to global breathlessness research [Box 1], followed by structured discussions to meet our aims.

Workshop attendees were provided with a proforma at the beginning of the workshop and listed their key topics for discussion. A further proforma was distributed after Day 1 requesting reflections. Preliminary conclusions and recommendations were agreed at the workshop. Consensus was reached during the editorial process and submission of the manuscript. Here we present key aspects of our discussions and recommendations arising from our workshop, to promote discussion—not to provide empirical evidence

Box 1 Workshop aims and topics

**Table:** 

#	Presentation topic	Aims
1	Breathlessness recognition, assessment and management in a high-income country (Where have we come from? Where are we now? Where are we going?)	On Day 1 to: 1) understand the current state of global breathlessness research and identify gaps; and 2) identify priorities and opportunities for global breathlessness research
2	Work of the International Primary Care Respiratory Group; identifying allies and advocacy	
3	Pulmonary rehabilitation research in South Asia	
4	Breathe-India—findings from a realist review and intervention co-design	
5	Global health and air pollution	On Day 2 to; 1) explore synergies between global breathlessness research and other aspects of global health; 2) understand how we can plan for, and conduct impactful global health research 3) identify how family-centred outcomes can contribute to the attainment of global health goals
6	Creating pathways to impact: lessons from global health research	
7	The role of informal carers in supporting people with advanced illness	
8	Social and economic burden of illness—insights from two projects conducted in Brazil	
Overall workshop aims: • To understand breathlessness in the broader context of global health • To develop an agenda for global breathlessness research

## We live in an increasingly breathless world

Until recently, estimations for the global burden of breathlessness were based on research conducted in high-income countries mostly through health service contact or extrapolated from disease prevalence data^5^. We discussed a recent cross-sectional study of 3046 community-dwelling adults in India, of which nearly half (1351, 44%), reported long-term *breathlessness limiting exertion* (modified Medical Research [mMRC] scale ≥1), with ~4% experiencing the most intense level of breathlessness (mMRC 4)^8^. In India, this represents 626 million people living with breathlessness of some intensity and 52 million people living with *such debilitating breathlessness that they are unable to leave the house or dress or undress without breathlessness*. This evidences the huge burden of breathlessness in an LMIC and indicates a much higher prevalence of breathlessness than in HICs. We explored reasons why breathlessness may be so prevalent—beyond known associations, focussing on sedentary behaviours and the influence of environmental factors.

Breathlessness is both a cause and a consequence of physical inactivity. Despite research that highlights this relationship, this issue has not yet been explored beyond advanced illness, despite increasing physical inactivity and rising obesity. We discussed how the environment may promote physical inactivity and breathlessness in LMICs. Heat, humidity, air pollution, traffic, lack of footpaths, time and other contextual factors make daily exercise a challenge—especially for women. For many, climate change is already making active lifestyles even more challenging.

We discussed the unknown relationship between air pollution and breathlessness prevalence. A recent study conducted in a single city in the United Kingdom identified how presentations to health services with respiratory problems increased during times of high air pollution^9^. The relative risks increased every 10 μg/m^3^ increase in NO_2_ and PM_2.5_ above the WHO recommended 24-h thresholds. The *highest* pollutant values recorded in the study were 80 μg/m^3^. In one area of Delhi, the *average* μg/m^3^ is 191.

Breathlessness may be a marker of unhealthy societies and environments. We urgently need to understand the structural reasons why breathlessness is so prevalent in LMICs. Doing so is essential for providing the context for global breathlessness research, how to reduce the burden and intervene to improve the lives of those affected.

**Recommendation 1.** Researchers in LMICs can measure the prevalence and consequences of long-term breathlessness in the general population at low cost using digital methods, to provide evidence to policymakers and clinicians of societal issues caused by long-term breathlessness (physical, financial, social, environmental, emotional, existential and sexual).

**Recommendation 2.** The causes of breathlessness need to be understood at societal levels. What is the relationship between breathlessness and air pollution? Is climate change worsening breathlessness and breathlessness-related outcomes?

## Developing a workforce to deliver family-centred care

Daily breathlessness which limits exertion occurs in adults of any age, generated by a range of lifestyle, environmental, disease and poverty-related factors^4^. Daily breathlessness may have multiple causes and many people living with daily breathlessness do not know its cause.

Breathlessness of unknown cause is a safety risk to patients and families because of delayed or inappropriate treatment *and* the impact of the symptom. Late, or lack of diagnoses increases excess mortality from illnesses causing chronic breathlessness^10^. The COVID-19 pandemic has also complicated understandings of breathlessness, with the increasing use of pulse oximeters re-inforcing misperceptions that breathlessness and ‘hypoxia’ are synonymous. The challenge of diagnosis and treatment alongside the need for symptomatic relief, makes breathlessness an excellent case study of the need for integrated approaches to provide patient-centred care.

Palliative care specialists in India report positive experiences of teaching breathlessness self-management strategies to their patients, their friends and family members. By contrast, other physicians describe *therapeutic helplessness* when faced with people who are breathless but without the skills to provide support^11^. Evidence that chronic breathlessness is a problem in the general population in LMICs, not only in the context of advanced illness, highlights the need for intervention at primary, secondary and community levels.

There is an urgent need for preventative, diagnosis, disease-modifying measures targeting underlying aetiologies. However, the immediate presence of debilitating, long-term breathlessness also requires urgent attention and should be considered a distinct therapeutic target. Unless in an emergency situation, diagnostic approaches will be complemented by breathlessness self-management education, which does not mask any underlying cause, but does provide symptomatic relief. If global health actors are serious about implementing integrated family-centred care, then symptom-based approaches must be taught and delivered alongside disease-modifying therapies as part of routine practice.

**Recommendation 3**. Contextually appropriate diagnostic pathways and management plans should be developed to support health workers at every level of a health system to distinguish between acute and long-term breathing problems, manage any reversible causes of breathlessness and actively address residual symptom burden.

## Breathlessness interventions; strength of evidence and the value of information

Immediately prior to our workshop, the European Respiratory Society (ERS) published Clinical Practice Guidelines on symptom management in serious respiratory illness summarising current evidence^12^. The ERS highlights low certainty of evidence and the modest impact of interventions on patient-centred outcomes and recommends new approaches to reduce symptoms and enhance wellbeing. They conclude that implementation of multi-component services may be more feasible in HICs where there is an established infrastructure and greater access to a multidisciplinary team. In contrast, the International Primary Care Respiratory Group (IPCRG) launched their ‘desktop helper,’ a suite of evidence-based breathlessness assessment and self-management techniques recommended for implementation by primary care health workers^12^. We discussed the implications of these developments for global breathlessness, concluding that, despite low certainty of the evidence for some breathlessness interventions, implementation, including hybrid approaches which combine evaluation and implementation measures, rather than efficacy research should be prioritised in LMIC—for two key reasons.

First, further evaluation using randomised trials of best-available breathlessness self-management is unlikely to modify greatly the interventions offered. Nor will further effectiveness evaluation address the ‘know-do’ gap—how to implement evidence into practice in LMICs. Many breathlessness self-management interventions reflect cultural practices developed in LMICs. Handheld fans do have an effectiveness evidence-base^13^ albeit imprecise, and are widely available around the world. There is clinical trial evidence that yoga^14^, Tai Chi^15^ and Qi Gong^16^ improve breathlessness-related outcomes. Implementation of these approaches as a medical intervention for breathlessness may require a cognitive shift but are culturally associated with health benefits in Asia indicating acceptability, feasibility and scalability of delivery. Projects such as the NIHR-funded Global RECHARGE programme highlight cultural practices in low-resource settings relevant to respiratory outcome improvements^17^. For example, RECHARGE identified in Kyrgyzstan how a local practice of ‘rhythmic movement’ in can be incorporated as part of breathing exercises to enhance acceptability^18^, in India, yoga was included for the same reason. For such interventions, given the potential for improvement of breathlessness, the Value of Information of further large effectiveness trials seems questionable, and GRADE recommendations should take care not to worsen existing inequities^19^.

Secondly, we considered the emphasis on multidisciplinary teams for delivering multicomponent breathlessness interventions in LMICs to be unhelpful. Our work with stakeholders in India to co-design a multi-component breathlessness intervention highlighted opportunities for the use of handheld fans, breathing exercises and paced physical activity. These intervention components can be delivered by clinicians or lay workers and do not require a multidisciplinary team. Safety, evidence of patient benefits and low cost of breathlessness self-management techniques create a strong mandate for implementation despite low-grade effectiveness evidence.

**Recommendation 4**. In LMICs, implementation research should be prioritised to:

Identify core components of effective multi-component breathlessness interventions; and

Develop strategies for effective and system-wide implementation including workforce roles and cultural adaptations.

**Recommendation 5.** Assessment of the strength of evidence should be supported by the development of a ‘feasibility for translation evaluation framework, including health workers’ time-needed to treat^20^, to support implementation in LMICs.

**Recommendation 6.** Implementation research in LMICs is conducted using robust methods (e.g. Type 2 hybrid implementation trials incorporating dual testing of clinical and implementation interventions/strategies) which addresses the ‘know-do’ gap in country contexts.

## Addressing and measuring meaningful family-centred outcomes

It is recognised that breathlessness interventions developed in HICs must be adapted for delivery in LMICs. There is less acknowledgement that outcome measures by studies conducted in HICs may not reflect family and societal priorities in LMICs. A recent global health funding call addressing multiple long-term chronic conditions encouraged the use of measures that focus on disability and health-related quality of life; clinically meaningful to people with daily breathlessness.

Breathlessness negatively influences workforce participation in HICs^21^. Qualitative research in Tanzania, reports serious challenges for those with respiratory diseases in conducting physical work, contributing to poverty and potentially premature mortality^22,23^. Given the high prevalence of daily breathlessness across all groups in India, the global economic impact of breathlessness *could* be staggering. The extent to which socioeconomic patient and family-centred outcomes are modifiable by breathlessness self-management interventions is unknown. Global research must go beyond health-related measures only, to address outcomes that reflect the lived experiences of patients and families, where unmanaged breathlessness may mean unemployment.

**Recommendation 7.** We urgently need to understand the global economic impact of daily breathlessness and the extent to which implementation of self-management interventions can alter socioeconomic outcomes for people living in societies with few social safety nets.

## Developing a language for global breathlessness research

Getting the language right is crucial for engaging with patients; reflecting their concerns and framing breathlessness to research funders and policymakers as a key issue for global health. *Dyspnea* is still used by clinicians and within outcome measures but is not meaningful to patients. Terms, like *chronic* or *refractory* breathlessness—are *intended* to distinguish long-term, persistent breathlessness from a normal physiological response to exertion, but have lay meanings of ‘severe’ and ‘hopeless’. Crucially, any definition that assumes that optimal management of underlying causes is feasible before treating breathlessness as a distinct therapeutic target does not promote an integrated approach. Stakeholders in India prefer the term ‘Daily, Distressing, Disabling Breathlessness,’ (shortened to Daily breathlessness) because it is simple and reflect the all-encompassing consequences of breathlessness (we note that in many world languages, there is no direct translation for ‘breathlessness’—and equivalent words often come with their own associations. For example, in Bangladesh, the word dam is used as a synonym for breathlessness, asthma, and COPD. Translation work and adaptation to context are necessary in all settings).

In addition to breathlessness-related terms, the language used for symptom-directed approaches also comes with its own connotations and may unintentionally undermine credibility. The use of terms such as ‘non-medical’ or ‘non-pharmacological’ was felt to create negative perceptions. Potentially even worse, is “self-management” approaches given that they are easily relegated to a secondary role, may indicate somehow that these are not cutting edge or effective or imply a level of ‘abandonment’ (do it yourself). Yet people living with daily breathlessness find self-management approaches helpful. We must work together to develop a language for breathlessness that empowers people to participate in their own care.

**Recommendation 8.** We promote **Daily, Distressing, Disabling Breathlessness (Daily Breathlessness**) as a person-centred language for breathlessness. Stakeholder engagement is necessary in LMICs to understand how to encourage people’s involvement in their own care (self-efficacy) using appropriate language.

## Concluding remarks

The global prevalence of breathlessness in LMICs may be much higher than previously estimated based on HIC data and anticipated relationships between disease burden and breathlessness. As breathlessness researchers and clinicians, we advocate for symptom-based approaches to be used in parallel to disease-modifying therapies. However, we have presumed that breathlessness is due to a medical condition for which treatment is available. Daily breathlessness persists for many despite the optimal management of their condition(s). Much more so where optimal management is unavailable, inaccessible, or where the condition(s)’ cause(s) may be an unavoidable context of daily life.

Breathlessness may be a flag for unhealthy environments and societies. A programme of global breathlessness research is required which addresses breathlessness at family and societal levels. At the family level, clinical research is important in the development and evaluations aimed at palliating the symptom. However, we must also make the best use of the current evidence through implementation studies in the LMIC context and by empowering communities. At the societal level, we must look beyond breathlessness as a clinical problem to develop new paradigms for understanding the causes of breathlessness and the consequences of living in societies with a high prevalence of long-term breathlessness. Can improving understanding of breathlessness and its consequences mobilise the knowledge necessary to address wider structural problems facing families, communities and societies?

We did not specifically address the issue of developing research capacity for global breathlessness research during our workshop, although it was an assumption based on prior work. Funding and a skilled workforce are urgently needed to deliver the programme of global breathlessness research we propose. The next step for the authorship team is to pursue funding so that we can further validate and act on our recommendations. However, multiple interdisciplinary teams and the support of global funding agencies will be needed to act on our recommendations. One of our collaborators, the International Primary Care Respiratory Group held a workshop at its 2023 Scientific Meeting on breathlessness in primary care and has fed into this publication looking at gaps in knowledge as well as current assets. One of its key findings was that breathlessness diagnosis and management requires clinicians to manage uncertainty due to the lack of evidence but also personal variability. Therefore it convened an expert group to write its Desktop Helper https://www.ipcrg.org/DTH17 which also identified areas of uncertainty that require research. The challenges include 1. lack of recognition by funders of the importance of breathlessness to healthcare utilisation and quality of life; solutions: papers such as this which show that breathlessness would be a productive avenue of multi-morbidity research given its frequency, complexity and low-cost solutions and sessions at scientific meetings such as that organised by IPCRG in 2024 (its own conference and WONCA Europe 2024); 2. The lack of consistent coding of symptoms in healthcare records; solution: encourage the use of symptom codes in primary care; 3.The need for multi-disciplinary research teams. Solution: create a network with physiotherapists, mental health, cardiology, respiratory, palliative care and primary care researchers. The IPCRG is already developing a network of interested partners in high, middle and low-income countries and this can be built on further. Some of the participants of the workshop were introduced by IPCRG. We invite study teams interested in critiquing or contributing to the agenda of global research we have outlined here to get in touch to discuss collaboration—especially people in LMICs. Global breathlessness research can only proceed with professional collaborations and meaningful involvement of community stakeholders.
